# Minor Surgery

**Published:** 1862-05

**Authors:** 


Minor Surgery. On Bandaging and other Operations of Minor Surgery. By F. W. Sargent, M.D., Member of the College of Physicians of Philadelphia, one of the Surgeons to Wills' Hospital, etc., etc. New Edition, with a Chapter on Military Surgery, By W. F. Atlee, M.D., and 187 Illustrations. Philadelphia: Blanchard & Lea. 1862.

This is just such a work as its title imports. The first edition has been some time before the profession and its merits are well known. The present edition is considerably enlarged and, with the chapter on Military Surgery, will be found exceedingly convenient, and valuable to all members of the profession; but more especially so, to Military Surgeons and Students. It is published in the well-known excellent style of Blanchard & Lea. 12mo., pp. 383.

